# Three *ABO* 3′ splice-site variants including a novel one converge on a common splicing defect to attenuate blood group antigen expression

**DOI:** 10.3389/fimmu.2026.1959480

**Published:** 2026-10-02

**Authors:** Hongli Guan, Yalan Yang, Jian Li, Huan Zhang, Xue Chen, Jialiang Gao

**Affiliations:** Blood Grouping Reference Laboratory, Chengdu Blood Center, Chengdu, Sichuan, China

**Keywords:** ABO subtypes, antigen expression, exon skipping, splice-site variants, transfusion

## Abstract

**Background:**

ABO subtypes are characterized by attenuated antigen expression, rendering them susceptible to serological misclassification and posing substantial risks to transfusion safety. Splice-site variants represent one of the mechanisms underlying ABO subtype formation; however, previous mechanistic investigations have predominantly focused on the 5′ splice-site(5′SS), leaving the functional consequences of 3′ splice-site (3′SS) variants largely unexplored.

**Methods:**

Serological phenotyping of a proband with ABO grouping discrepancy was performed using tube agglutination, microcolumn gel agglutination, and absorption-elution assays. Genotyping was resolved by long-range PCR coupled with Nanopore sequencing. The splicing impact of this novel variant and two previously reported variants, c.29-3C>G and c.29-10T>A, was predicted *in silico* and validated by *in vitro* minigene assay. Computational saturation mutagenesis was further deployed to systematically delineate a preliminary framework of regulatory principles governing 3′SS variation.

**Results:**

A novel 3′SS variant, c.29-2A>G, was identified in an ABO*A1.02 background in a proband with an A_el_ phenotype. This novel variant and two previously reported variants disrupted the canonical 3’SS of intron 1, converging on exon 2 skipping as the predominant aberrant splicing event. Additionally, c.29-10T>A activated a cryptic acceptor site, generating a secondary transcript with an 8-bp intronic retention. All aberrant transcripts disrupt the open reading frame and introduce premature termination codons. Through computational saturation mutagenesis, we further established an initial position- and base-dependent risk stratification model for 3′SS variants.

**Conclusion:**

This study provides the first direct mechanistic evidence linking *ABO* 3′SS variants to subtype pathogenesis, demonstrating that exon 2 skipping constitutes a shared molecular lesion across intron 1 3′SS variants, ultimately leading to attenuated blood group antigen expression. The preliminary computationally derived risk stratification framework established herein offers a clinically valuable reference for interpreting 3′SS variants, with implications that extend beyond blood group genetics to the broader landscape of splicing-mediated human diseases.

## 1 Introduction

The ABO blood group system, discovered by Landsteiner in 1900, remains the most clinically significant human blood group, with importance extending beyond transfusion to transplantation and forensics (1–3). The synthesis of A or B antigens on erythrocytes is catalyzed by the glycosyltransferase A or glycosyltransferase B, respectively, both of which are encoded by the *ABO* gene at 9q34.2 (4). Genetic variants in the *ABO* gene can alter glycosyltransferase function, giving rise to a spectrum of ABO subtypes characterized by attenuated antigen expression (5). Among these, the A_el_ phenotype represents a particularly extreme example, defined by markedly diminished A antigen levels that are insufficient to produce direct agglutination with anti-A or anti-A,B antibodies, yet remain detectable by absorption-elution tests (6, 7). Clinically, such weak expression renders A_el_ individuals prone to serological misclassification, and the accompanying naturally occurring anti-A_1_ antibodies place them at risk of hemolytic transfusion reactions if transfused with incompatible blood (8). Accurate identification of such subtypes via genotypic and mechanistic characterization is therefore fundamental to diagnostic reliability and transfusion safety.

Currently, over 400 ABO variants have been documented, with the vast majority residing in exonic regions and a minority located in noncoding regions, including intronic regulatory elements and splice sites (9). Despite their relative rarity, splice-site variants are of considerable clinical and biological importance, as proper pre-mRNA splicing relies on accurate exon-intron junction demarcation, achieved by the spliceosome through recognition of the 5′SS and 3′SS (10). In this process, the 5′SS directs spliceosome assembly at the upstream boundary, while the 3′SS governs acceptor recognition and exon ligation (11, 12). Specifically, the 3’SS is defined through coordinated binding of Splicing Factor 1 (SF1) to the branch site and U2 small nuclear RNA auxiliary factor 2 (U2AF2) to the polypyrimidine tract, which together direct U2 small nuclear ribonucleoprotein (snRNP) recruitment and subsequent AG-dependent exon ligation (13–16). Given the precision of these recognition events, disruption by splice-site variants can occur via diverse mechanisms that ultimately lead to aberrant mRNA transcripts (17, 18). Previous mechanistic investigations into splice-site variations underlying the ABO phenotypes have largely focused on the 5′SS (19–22), whereas the contribution of 3′SS variants remains considerably unexplored. This skewed distribution may be partly attributable to the fact that 3′SS variants are inherently less frequent than 5′SS variants in human genes (23).

Third-generation sequencing (TGS), with its long-read advantage, enables full-length haplotype sequencing of the *ABO* gene, providing comprehensive coverage of coding regions, introns, and flanking regulatory sequences, thereby facilitating the detection of intronic variants that were previously challenging to identify with conventional approaches (24). Using TGS, we identified a novel 3′ splice-site variant, c.29-2A>G, in intron 1 of the ABO*A1.02 allele from a proband presenting with A_el_ phenotype. Of note, four distinct variants have been reported in close proximity to the intron 1 3′SS of the ABO gene: c.29-3C>G on ABO*B.01 (25), c.29-5T>G on ABO*A1.01 (26), c.29-10T>A (27), and c.29-10T>G on ABO*A1.02 (28). All four variants uniformly impair ABO antigen expression, giving rise to ABO subgroup phenotypes. Except for c.29-5T>G in ABO*A1.01, which exhibits mild inter-individual serological variability, the remaining three variants are consistently associated with the B_el_ or A_el_ phenotypes (24, 25). These findings suggest that such variants may converge on a shared splicing defect, ultimately leading to barely detectable antigen expression. To validate this hypothesis, we selected c.29-3C>G, c.29-10T>A, and our newly identified c.29-2A>G variant as representative 3′SS variants for functional characterization. We integrated *in silico* splicing predictions with *in vitro* minigene assays to dissect the molecular basis of these three 3′SS variants. Furthermore, computational saturation mutagenesis was employed to assess the impact of single-base substitutions at distinct positions on acceptor site stability, revealing shared regulatory signatures among these variants. Collectively, this study aimed to provide the first mechanistic evidence linking 3′SS variants to ABO phenotypes and establish a preliminary systematic framework for deciphering the regulatory logic governing splicing at this locus.

## 2 Methods

### 2.1 Peripheral blood sampling

The proband was a healthy individual of Chinese descent whose initial ABO grouping revealed discordant forward and reverse typing results. Exclusion criteria comprised a history of blood transfusion, hematopoietic stem cell transplantation, or other inherited disorders that could interfere with ABO antigen expression. Following written informed consent, 2 mL of peripheral venous blood was obtained and collected into an EDTA-containing tube.

### 2.2 ABO blood grouping

ABO phenotyping of the proband was performed in parallel using the standard tube agglutination test and the gel column agglutination test. For the tube test, forward grouping employed anti-A, anti-B, anti-A,B, and anti-H reagents (Shanghai Hemo-pharmaceutical Biological Company), while reverse grouping was carried out using A_1_, B, O, and A_2_ reagent erythrocytes (Shanghai Hemo-pharmaceutical Biological Company) for plasma antibody identification. For the gel card assay, testing was conducted with blood grouping gel cards (Bio-Rad, Switzerland) strictly in accordance with the manufacturer’s instructions, with reverse grouping employing A_1_ and B standard erythrocytes (Shanghai Hemo-pharmaceutical Biological Company). To confirm weak A antigen expression, an adsorption-elution assay was additionally performed using human-derived anti-A serum (Shanghai Hemo-pharmaceutical Biological Company), following the protocols specified in the AABB Technical Manual (20th edition).

### 2.3 *ABO* genotyping by long-range PCR and nanopore sequencing

The *ABO* gene haplotype of the proband was resolved using a three-fragment long-range PCR strategy with three overlapping primer pairs, generating three adjacent amplicons (9.0, 9.6, and 12.0 kb) sharing ~1.3-kb overlaps and covering the entire *ABO* locus. After library preparation, the amplicons were sequenced on the PolyseqOne platform. Following basecalling and demultiplexing (PolyseqSequence), reads were aligned to the T2T-CHM13v2.0 reference genome for *ABO* genotyping and variant calling (minimap2, v2.28-r1209). Detailed PCR conditions, library preparation, and bioinformatic analysis parameters have been described in our previous study on a 5′SS splice-site variant (29).

### 2.4 *In silico* splicing analysis of three *ABO* intron 1 3′SS variants

To explore how intron 1 3′SS variants perturb pre-mRNA splicing, we analyzed c.29-2A>G and two previously reported variants (c.29-3C>G and c.29-10T>A) in the ABO*A1.02 background (25, 27). Although originally identified on a B.01 allele, c.29-3C>G was evaluated in this background to rule out allelic confounders. Splicing patterns were predicted using RDDC (https://rddc.tsinghua-gd.org/zh), and quantitative scores for sites S1–S5 (**Figure 1A**) were obtained using SpliceRover (http://bioit2.irc.ugent.be/rover/splicerover), NetGene2 (https://services.healthtech.dtu.dk/service.php?NetGene2-2.42), and ASSP (http://wangcomputing.com/assp/index.html).

**Figure 1:**
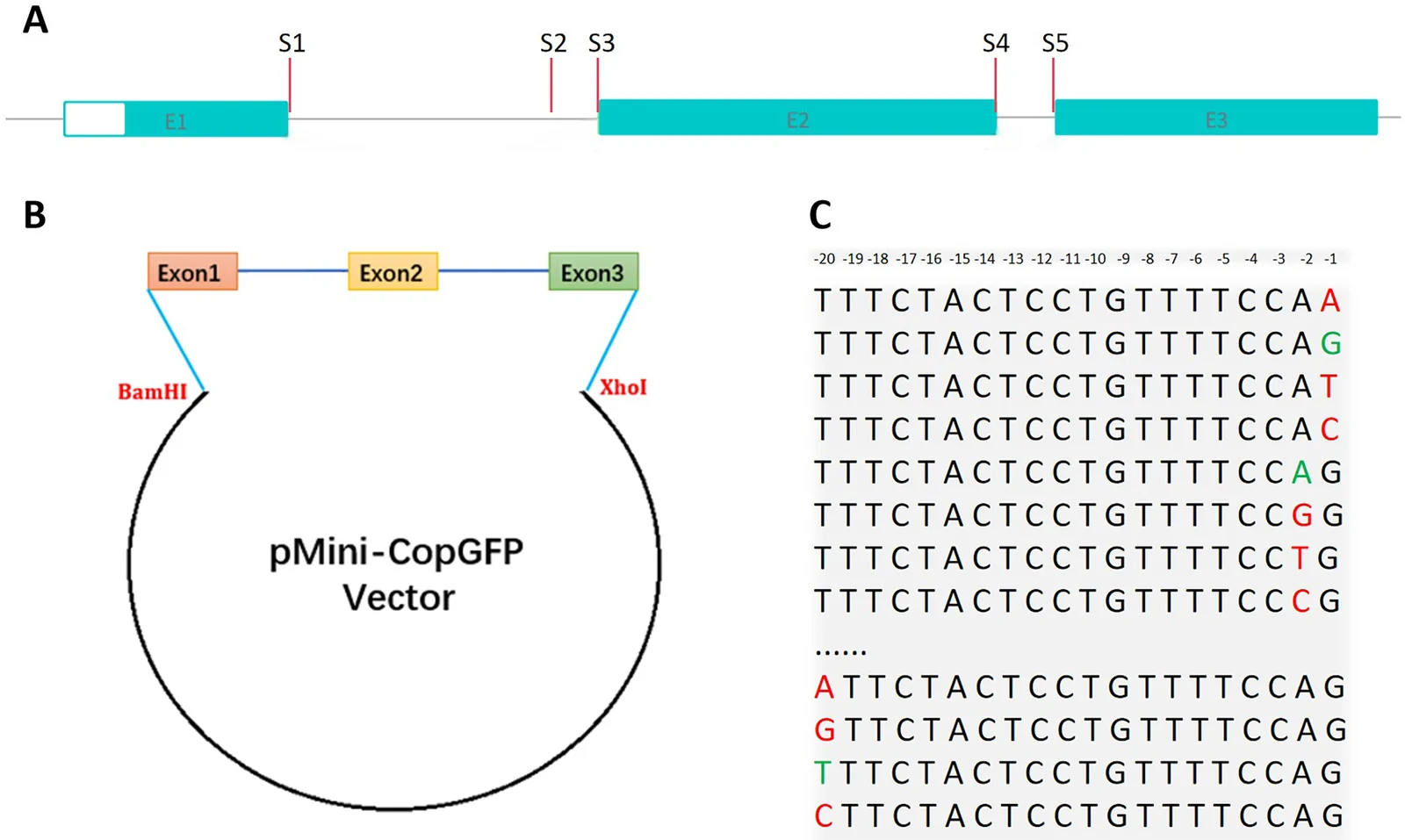
**(A)** Schematic of the partial ABO genomic locus (exons E1-E3 and flanking introns). S1/S4, canonical 5’ donors; S3/S5, canonical 3’ acceptors; S2, predicted alternative 3’ acceptor. **(B)** Schematic of the minigene constructs. The wild-type and three mutant constructs were generated by fragment assembly or site-directed mutagenesis. **(C)** Computational design of saturation mutagenesis library spanning the 20-nt region upstream of the intron 1 3′ acceptor. Wild-type, green; substituted, red.

### 2.5 Minigene splicing assay

Minigene splicing assays were performed to validate the in silico predictions. To obtain the insert, two fragments were amplified using two pairs of primers: one covering exon 1 and its downstream intronic region, and the other spanning the mutation-bearing intronic region to the 5′ end of exon 3 (**Supplementary Table 1**). These two amplicons were subsequently assembled to reconstruct the complete insert. Using genomic DNA from a healthy control and the proband as templates, the wild-type and c.29-2A>G inserts were obtained, respectively, and cloned into the pMini-CopGFP vector via the BamHI and XhoI sites to generate the wild-type plasmid and the c.29-2A>G construct (**Figure 1B**). For the two previously reported variations, c.29-3C>G and c.29-10T>A, site-directed mutagenesis was performed using the wild-type plasmid as a template and primers carrying the desired substitutions (**Supplementary Tables 2, 3**). All resulting constructs were verified by Sanger sequencing prior to functional assays. HEK293T cells were selected for transient transfection. Compared with the K562 cell line (30), HEK293T cells offer a simpler experimental workflow and higher transfection efficiency. Moreover, HEK293T cells possess the full complement of splicing machinery required for accurate pre-mRNA processing, ensuring reliable detection of variant-induced aberrant splicing events (31). The pMini-CopGFP vector was chosen for its hPGK promoter, which provides moderate transcription levels suitable for splicing assays, as well as its built-in quality-control intron and independent CopGFP reporter for monitoring transfection efficiency. This pMini-CopGFP/HEK293T system was validated in our previous ABO splice-site functional study (29). All subsequent procedures, including cell transfection, total RNA extraction, RT-PCR (primers listed in **Supplementary Table 4**), and the analysis of the resulting splicing products by agarose gel electrophoresis, capillary electrophoresis, and Sanger sequencing, were performed as described previously.

### 2.6 Computational saturation mutagenesis of the *ABO* intron 1 3′SS upstream region

For a systematic dissection of the regulatory impact of single-base substitutions at different positions on native acceptor site (S3) stability, computational saturation mutagenesis was performed by substituting A/G/T/C at each position1 to20 upstream of the acceptor site in the ABO*A1.02 background (**Figure 1C**) (32). Splicing pattern prediction and S3 score acquisition were performed using the same methods as above (RDDC, ASSP, NetGene2, and SpliceRover) with the predicted scores visualized as bar charts and line graphs. To assess the relationship between positional distance and splicing scores while controlling for base identity, we selected positions where T was the wild-type base (10 of 20 positions) for correlation analysis. One position (10 T>A), corresponding to the c.29-10T>A variant, was identified as an extreme outlier for SpliceRover regression, based on its outlying scores (**Figure 2B**), and was excluded prior to modeling. Simple linear regression was performed using GraphPad Prism 11, and the results were presented as scatter plots with regression lines.

**Figure 2:**
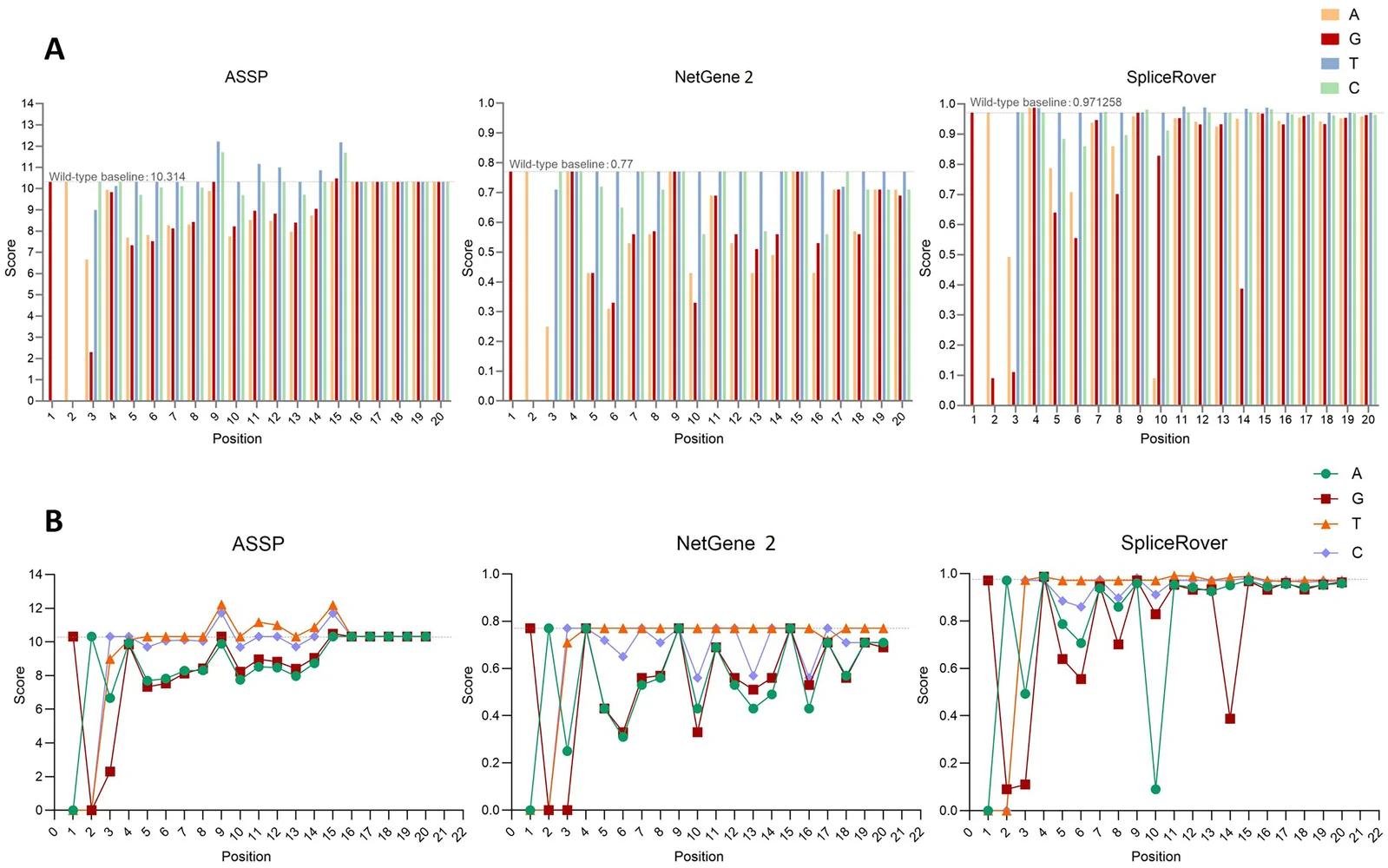
*In silico* prediction of 3′ splice sites based on a computational saturation mutagenesis model of ABO intron 1. **(A)** Bar plots showing ASSP, NetGene, and SpliceRover scores for A/G/T/C substitutions at positions 1-20. **(B)** Line plots of score profiles across the same positions. In all panels, the X-axis denotes the distance to the 3’SS (bp), and the Y-axis shows tool-specific prediction scores. Horizontal dashed lines indicate the wild-type baseline.

## 3 Results

### 3.1 Serological results

The ABO blood group serology results for the proband are summarized in **Table 1**. Tube testing showed no reactivity with anti-A, anti-B, or anti-A,B, but strong (4+) reactivity with anti-H; reverse typing revealed 1+^w^ agglutination with A_1_ cells, 2+^s^ with B cells, and no agglutination with O or A_2_ cells. Gel column agglutination was consistent, showing no forward reactivity with anti-A or anti-B; reverse typing yielded 2+ agglutination with A_1_ cells, 4+ with B cells and no reaction in the control. Critically, adsorption-elution confirmed weak A antigen expression, with the eluate showing 1+^w^ reactivity with A_1_ cells but no reactivity with B or O cells. Collectively, these findings are consistent with an A_el_ phenotype accompanied by naturally occurring anti-A_1_.

**Table 1:** Serologic and molecular characteristics of the proband.

Forward typing (tube method)				Forward typing (gel column)		Reverse typing (tube method)				Reverse typing (gel column)			Adsorption and elution	Pheno-type	Genotype
Anti-A	Anti-B	Anti-A,B	Anti-H	Anti-A	Anti-B	A_1_c	Bc	Oc	A_2_c	A_1_c	Bc	Ctl	A_1_c		
0	0	0	4+	0	0	1+^W^	2+^S^	0	0	2+	4+	0	1+^W^	A_el_	ABO*A1.02(c.29-2A>G)/ ABO*O.01.02

W , weak reaction; ^S^, strong reaction; 0, no agglutination.

### 3.2 Single-molecule nanopore sequencing results

Nanopore sequencing revealed a single-base substitution(c.29-2A>G)disrupted the invariant AG dinucleotide of the intron 1 acceptor splice site on the background of an ABO*A1.02 haplotype (**Figure 3**). This variant was identified with a mean sequencing depth of 620× and 100% coverage of the target amplicon. The variant allele frequency (VAF) was 7/10,000,000 in gnomAD(Genome Aggregation Database) and 1/100,000 in ExAC (Exome Aggregation Consortium). No other exonic or intronic variants were detected within this haplotype. The proband carried a novel ABO*A1.02-like allele, defined by the c.29-2A>G substitution, along with an ABO*O.01.02 allele.

**Figure 3:**
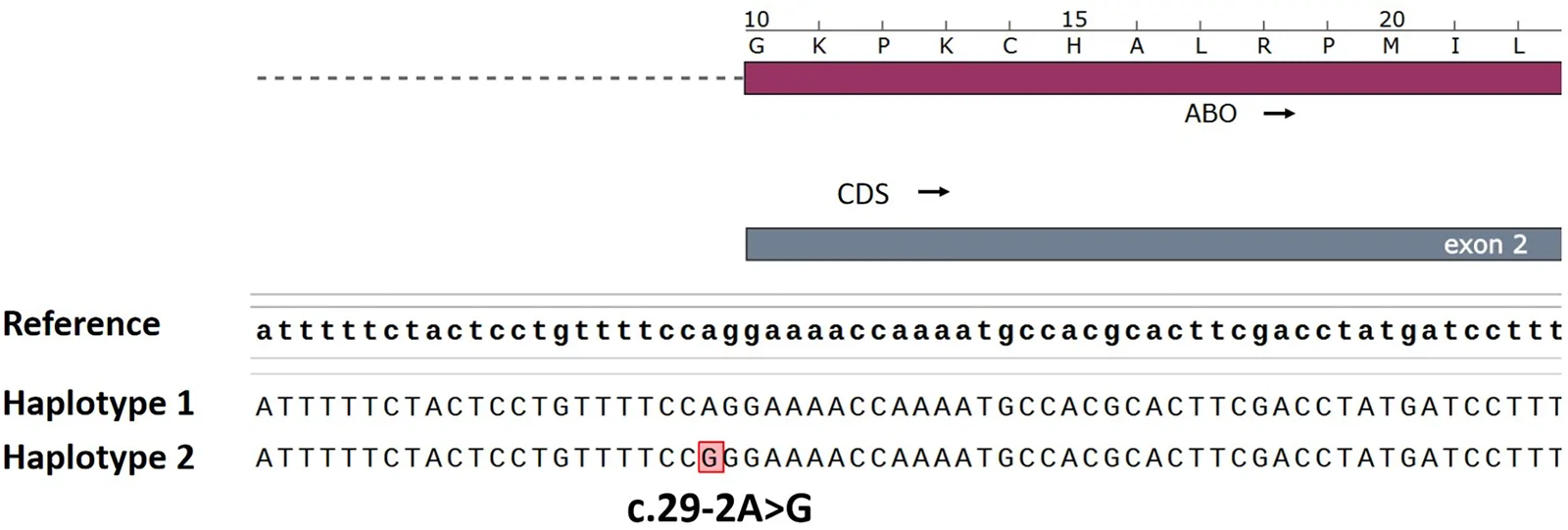
Nucleotide sequences flanking the c.29-2A>G variation in the proband, visualized using SnapGene v8.0.2.

### 3.3 *In silico* splicing predictions for three *ABO* intron 1 3’SS variants

#### 3.3.1 Predicted splicing patterns

The RDDC RNA Splicer tool uniformly predicted exon 2 skipping (a 70-bp deletion) for all three variants. An additional aberrant transcript with an 8-bp insertion was predicted exclusively for c.29-10T>A, attributed to the activation of a novel acceptor site (**Figure 4**).

**Figure 4:**
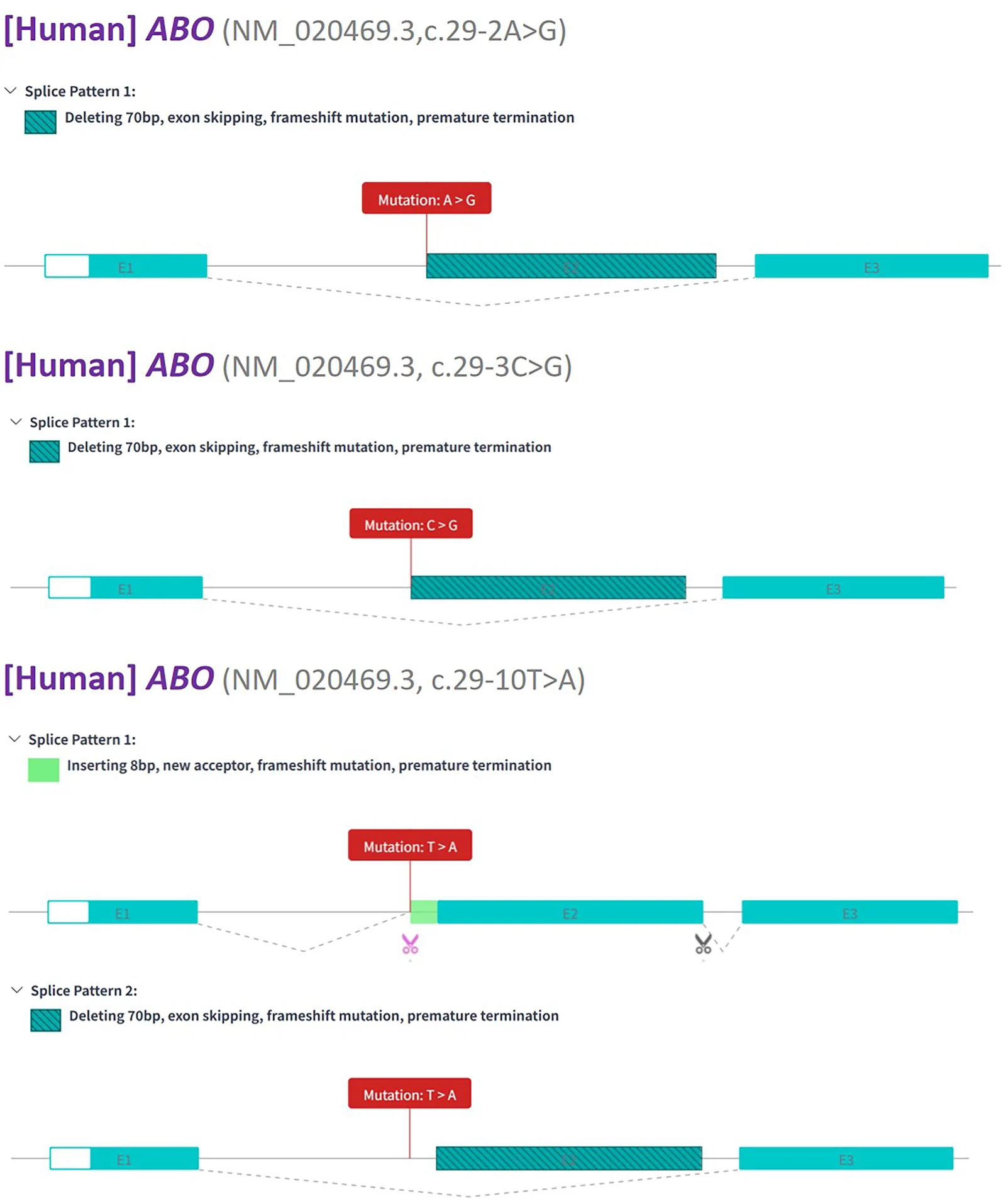
RDDC RNA Splicer prediction of the c.29-2A>G, c.29-3C>G, and c.29-10T>A variants. All three are predicted to cause exon 2 skipping (70-bp deletion), with an additional 8-bp insertion exclusively for c.29-10T>A via a novel acceptor site activation. All aberrant transcripts result in frameshift and premature termination.

#### 3.3.2 Splice-site prediction scores

**Table 2** summarizes the prediction scores from three tools. The five splice sites are depicted in **Figure 1A**, where S1 and S4 represent canonical 5’ donors; S3 and S5 represent canonical 3’ acceptors; and S2 represents a predicted alternative 3’ acceptor. ASSP scored the wildtype S3 as a strong constitutive acceptor (10.314); this signal was completely lost in c.29-2A>G, weakened to 2.299 in c.29-3C>G, and shifted to 7.750 in c.29-10T>A. Notably, an S2 signal (9.955) was detected exclusively in c.29-10T>A. NetGene2 yielded similar results: the wild-type S3 score (0.7) decreased to 0.43 in c.29-10T>A, whereas no S3 signal was detected for c.29-2A>G or c.29-3C>G. Consistently, an S2 signal (0.51) was observed only for c.29-10T>A. SpliceRover similarly demonstrated marked S3 score reductions and moderate S4 decreases, with c.29-2A>G, c.29-3C>G, and c.29-10T>A showing reductions from 0.971258 to 0.090461, 0.11058, and 0.090461, respectively, and from 0.611193 to 0.361094, 0.462848, and 0.553425 at S4. Again, an S2 signal (0.771202) was detected exclusively in c.29-10T>A. Scores at S1 (13.372) and S5 (9.983) remained unchanged relative to the wild-type across all tools.

**Table 2:** Splice-site prediction scores for wild-type and three 3' ss variants in intron 1 via SpliceRover, NetGene and ASSP.

Prediction tool	Variant	S1 (GGCCGGT^GAGTG)	S2 (ACTCCAG^TTTTTC)	S3 (TTTCCAG^GAAAAA)	S4 (TTTGGGT^AAGAC)	S5 (TCTGCAG^TTACG)
ASSP	WT	13.372	—	10.314	9.437	9.983
	c.29-2A>G	13.372	—	—	9.437	9.983
	c.29-3C>G	13.372	—	2.299	9.437	9.983
	c.29-10T>A	13.372	9.955	7.750	9.437	9.983
NetGene 2	WT	0.99	—	0.77	0.35	0.29
	c.29-2A>G	0.99	—	—	0.35	0.29
	c.29-3C>G	0.99	—	—	0.35	0.29
	c.29-10T>A	0.99	0.51	0.43	0.35	0.29
SpliceRover	WT	0.999453	—	0.971258	0.611193	0.772985
	c.29-2A>G	0.999453	—	0.090461	0.361094	0.772985
	c.29-3C>G	0.999453	—	0.110580	0.462848	0.772990
	c.29-10T>A	0.999453	0.771202	0.090461	0.553425	0.772985

WT, ABO*A1.02 allele; —, no detectable splice signal. ASSP scores refers to the integrated log-likelihood score assigned by the pre-processing models, where default cutoffs are 2.2 (acceptor) and 4.5 (donor); higher scores reflect stronger splice site motif conservation. SpliceRover and NetGene2 scores range from 0 to 1, with higher scores indicating stronger splice site propensity.

### 3.4 Minigene assay validation of three *ABO* intron 1 3’SS variants

#### 3.4.1 Fragment length analysis of minigene products

Gel electrophoresis of RT-PCR products revealed that the wild-type (WT) construct generated a single 238 bp product (comprising 71 bp of vector sequence, 40 bp of partial exon 1, 70 bp of exon 2, and 57 bp of exon 3). All three mutant constructs produced a shorter 168 bp band, with c.29-10T>A additionally yielding a longer 246 bp band (**Figure 5A**). Capillary electrophoresis further confirmed these results, showing a 236.29 bp fragment for the WT, a 167.85 bp fragment for both c.29-2A>G and c.29-3C>G, and both 167.85 bp and a minor 245.19 bp transcript in c.29-10T>A; no normal transcript was detected in any of the three mutant groups (**Figure 5B**).

**Figure 5:**
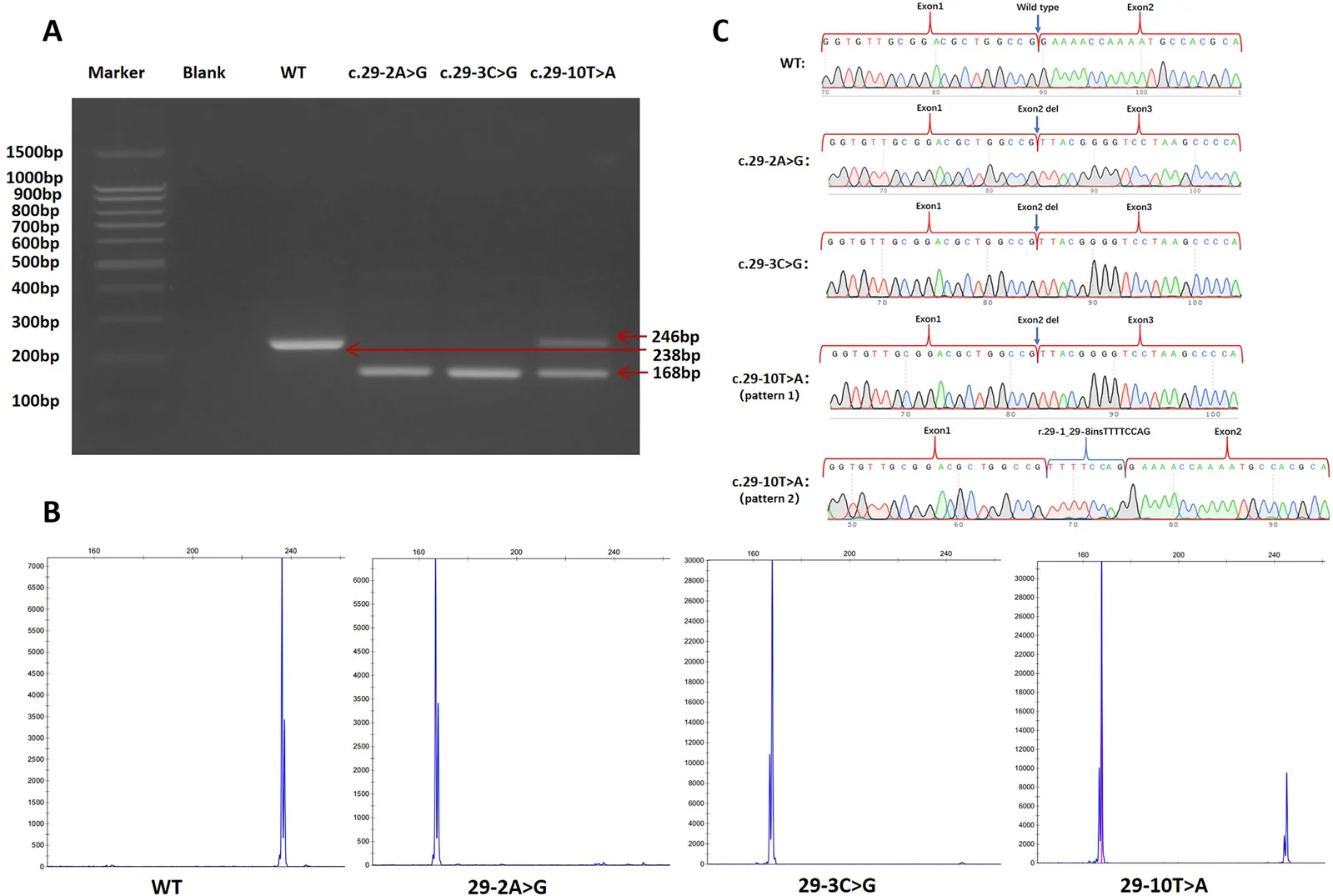
Minigene splicing assays. **(A)** Gel electrophoresis: WT yields a 238 bp band; c.29-2A>G and c.29-3C>G show a 168 bp band; c.29-10T>A produces two bands (168 bp and 246 bp). **(B)** Capillary electrophoresis: WT shows a 236.29 bp fragment; c.29-2A>G and c.29-3C>G show a 167.85 bp fragment; c.29-10T>A shows a 167.85 bp fragment and a small amount of 245.19 bp transcript. Percentages from peak areas (x: bp; y: RFU). **(C)** Sanger sequencing: all variants cause exon 2 skipping; c.29-10T>A additionally generates an 8-bp intronic insertion (c.29-1_29-8).

#### 3.4.2 Minigene product sequencing and splicing mechanistic analysis

Sanger sequencing confirmed normal splicing in the WT construct, while all three mutants exhibited an aberrant transcript resulting from exon 2 skipping, with c.29-10T>A additionally producing a transcript retaining an 8-bp intronic sequence (**Figure 5C**). Mechanistically, all three variants disrupted the canonical acceptor splice site at the 3′ end of intron 1, leading to exon 2 skipping and a 70-bp deletion; c.29-10T>A additionally activated a novel acceptor site, resulting in an 8-bp intron retention (**Supplementary Figure 1**).

### 3.5 Computational saturation mutagenesis on *ABO* intron 1 3’SS prediction analyses

#### 3.5.1 Splicing patterns induced by saturation mutagenesis

RDSS predictions (**Table 3**) revealed that not all variants disrupted the original splicing pattern. For instance, outside the conserved 1 to 3 region, T substitutions consistently preserved wild-type splicing. Likewise, at position 15, all tested substitutions also preserved wild-type splicing. As for all splicing-disruptive variants, exon 2 skipping was shared as a common aberrant event, with some variants additionally exhibiting other splicing abnormalities, such as activation of a novel acceptor site or alternative donor site.

**Table 3:** Summary of RDDC splicing outcomes induced by Computational saturation mutagenesis.

Position	A	G	T	C
1	New acceptor del 1bp+ Exon skip del 70bp	WT	Exon skip del 70bp	Exon skip del 70bp
2	WT	Exon skip del 70bp	Exon skip del 70bp	Exon skip del 70bp
3	Exon skip del 70bp	Exon skip del 70bp	Exon skip del 70bp	WT
4	Alt donor insert 152bp+ Consistent with WT+ Exon skip del 70bp	Consistent with WT	Consistent with WT	WT
5	Exon skip del 70bp	Exon skip del 70bp	WT	Alt donor insert 152bp+ Exon skip del 70bp
6	Exon skip del 70bp	Exon skip del 70bp	WT	Alt donor insert 152bp+ Exon skip del 70bp
7	Exon skip del 70bp	Exon skip del 70bp	WT	Exon skip del 70bp
8	Alt donor insert 152bp+ Exon skip del 70bp	Exon skip del 70bp	WT	Exon skip del 70bp
9	Alt acceptor insert 431bp+ Consistent with WT+ Exon skip del 70bp	WT	Consistent with WT	Consistent with WT
10	New acceptor insert 8bp+ Exon skip del 70bp	Exon skip del 70bp	WT	Exon skip del 70bp
11	Alt acceptor insert 431bp+ Consistent with WT+ Exon skip del 70bp	Alt acceptor insert 431bp+ Consistent with WT+ Exon skip del 70bp	Consistent with WT	WT
12	Exon skip del 70bp	Exon skip del 70bp	Consistent with WT	WT
13	Exon skip del 70bp	Exon skip del 70bp	WT	Exon skip del 70bp
14	Alt acceptor insert 431bp+ Consistent with WT+ Exon skip del 70bp	New donor del 105bp+ Exon skip del 70bp	Consistent with WT	WT
15	WT	Consistent with WT	Consistent with WT	Consistent with WT
16	Exon skip del 70bp	Exon skip del 70bp	WT	Alt acceptor insert 431bp+ Consistent with WT+ Exon skip del 70bp
17	Exon skip del 70bp	Alt acceptor insert 431bp+ Consistent with WT+ Exon skip del 70bp	Consistent with WT	WT
18	Exon skip del 70bp	Exon skip del 70bp	WT	Exon skip del 70bp
19	Exon skip del 70bp	Exon skip del 70bp	WT	Exon skip del 70bp+ Consistent with WT
20	Alt acceptor insert 431bp+ Exon skip del 70bp	Alt acceptor insert 431bp+ Exon skip del 70bp	WT	Alt acceptor insert 431bp+ Consistent with WT+ Exon skip del 70bp

Position,the distance (bp) to 3'SS;WT,the wild-type base at that position; Consistent with WT,a nucleotide substitution without altering the wild-type splicing pattern.

#### 3.5.2 Position- and base-dependent effects on splice-site prediction scores

Prediction score distributions from ASSP, NetGene, and SpliceRover are presented as bar plots in **Figure 2A**. Mutations at proximal positions 1 and 2 nearly eliminated canonical acceptor scores; position 3 displayed marked base-dependent score heterogeneity; position 4 exhibited strong mutational tolerance, with scores remaining close to baseline. Substantial score variability emerged between positions 5 and 14, whereas scores from ASSP and SpliceRover gradually returned to baseline levels in the distal 15 to 20 region. Splicing score trajectories (**Figure 2B**) further revealed synchronized fluctuation profiles for pyrimidines (T/C) and purines (A/G) across positions 5 to 14, a trend most evident in ASSP outputs. T scores were consistently at or above baseline, slightly higher than those of C; A and G followed broadly similar trends but displayed pronounced troughs at multiple loci and yielded lower values overall. This position- and base-dependent stratification was consistent with the splicing patterns summarized in **Table 3**.

#### 3.5.3 Correlation between positional distance and splicing scores

Linear regression on 10 wild-type T loci (**Figure 6**) revealed that T>A and T>G scores from all three tools were positively correlated with positional distance, with high goodness-of-fit across models (T>A: *R²* = 0.8140, *P* = 0.0004; T>G: *R²* = 0.9119, P < 0.0001). Position −10 was excluded as an extreme outlier from the SpliceRover T>A regression. For T>C variants, only ASSP and SpliceRover yielded statistically significant correlations (ASSP: *R²* = 0.4312, P = 0.0392; SpliceRover: *R²* = 0.5197, P = 0.0186), whereas NetGene showed no significant linear relationship (*R²* = 0.008392, P = 0.8013).

**Figure 6:**
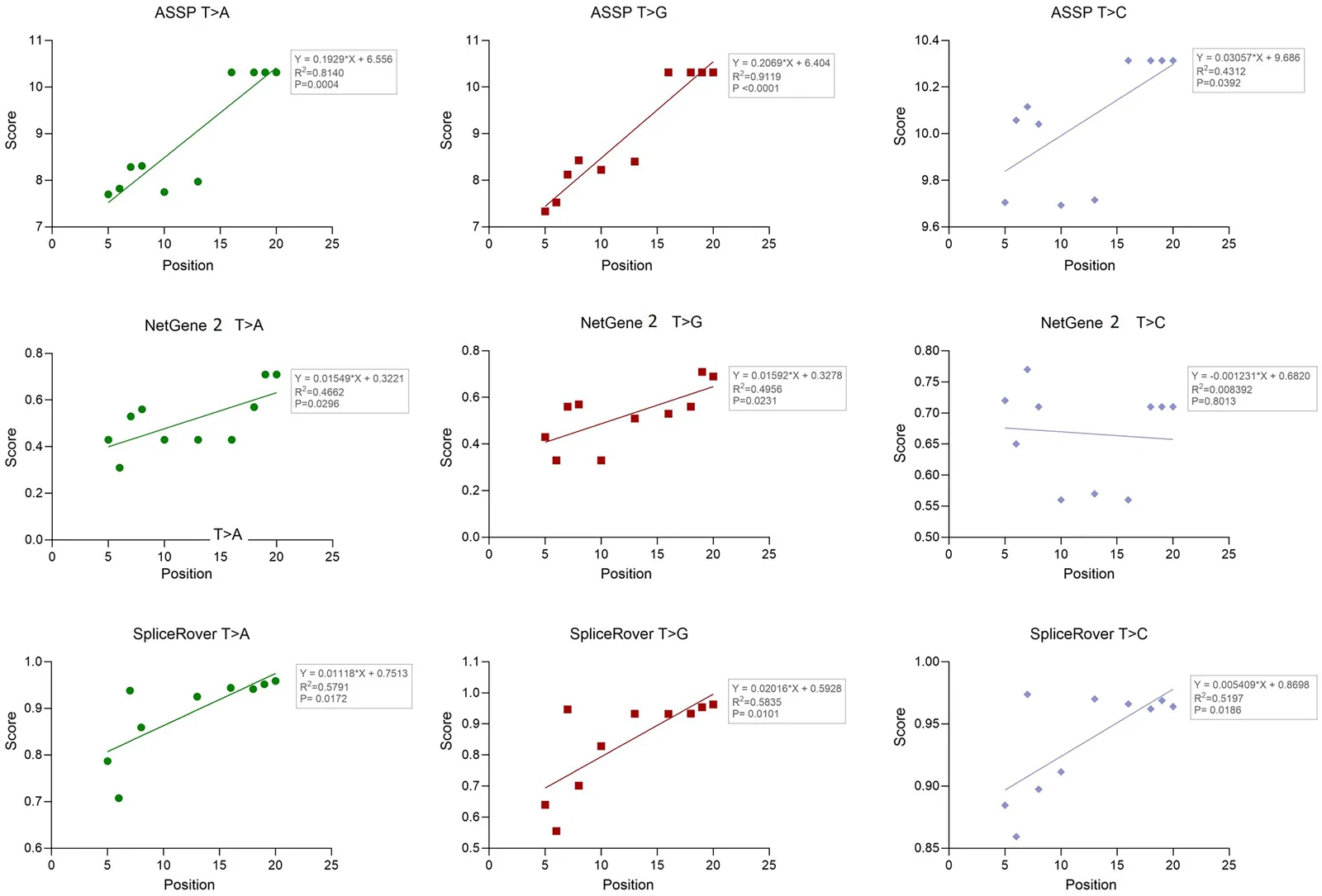
Linear regression of prediction scores versus distance to the 3’SS. X-axis: distance to 3′SS (bp); Y-axis: prediction scores. R²: goodness-of-fit (values approaching 1 indicate stronger linear relationship). P-values: significance (P < 0.05). Slope: direction and magnitude of the trend.

## 4 Discussion

ABO subtypes are inherited variants of the ABO blood group system characterized by diminished antigen expression (33). This serological profile often leads to misinterpretation in routine typing, creating a potential for hemolytic transfusion reactions (34). Therefore, accurate identification of ABO subtypes, coupled with mechanistic elucidation of antigen weakening, is essential for establishing a regulatory framework for antigen modulation and has direct implications for transfusion safety.

In this study, we identified a novel 3’SS variant, c.29-2A>G, on an ABO*A1.02 allele. This variant has not been deposited in the ISBT database (35) nor previously reported in the literature. The corresponding serological phenotype was a classic A_el_ phenotype, with concomitant anti-A_1_.

Pre-mRNA splicing is a fundamental process in eukaryotic gene expression, whereby introns are removed and exons are ligated to generate mature mRNA (36). Accurate 5’ and 3’ splice site recognition is essential for splicing fidelity, with the 5’ site directing spliceosome assembly and the 3’ site governing acceptor recognition and exon ligation (37, 38). Variants near either splice site can disrupt normal splicing at the canonical splice site, resulting in exon skipping or cryptic splice-site activation (39). These aberrant splicing events produce abnormal transcripts, ultimately giving rise to proteins that are either partially functional or completely nonfunctional (40). Although splice-site variations have garnered increasing attention in the molecular characterization of ABO subtypes in recent years, mechanistic studies have largely concentrated on 5’ splice-site variants (19, 29). However, 3’SS variations are rarely documented, and mechanistic studies on them remain scarce to date.

To address this critical gap, we performed parallel functional characterization of the novel c.29-2A>G variant alongside two previously reported 3’SS variations (c.29-3C>G and c.29-10T>A) (25, 27). Although c.29-3C>G was initially identified on a *B.01* allele, it was assessed here in the ABO*A1.02 background to rule out confounding effects from allelic differences. Integrating in silico predictions with a minigene assay, we elucidated the aberrant splicing mechanisms of the three variants.

The RDDC predicted that all three variants disrupt the canonical acceptor site (S3) and induce exon 2 skipping, with the c.29-10T>A variant additionally triggering activation of a cryptic acceptor site (S2) that generates an 8-bp intronic insertion (**Figure 4**). Consistently, prediction scores from SpliceRover, NetGene2, and ASSP corroborated these findings, showing marked reductions at the S3 site for all three mutants compared with the WT, along with detectable signals at S2 across all three tools (**Table 2**). These in silico predictions were experimentally validated by minigene assays, with both agarose gel electrophoresis and capillary electrophoresis of RT-PCR products from transfected minigene constructs confirming the 70-bp deletion transcript in all three mutants, and the c.29-10T>A construct additionally yielding a transcript carrying an 8-bp insertion (**Figures 5A, B**). Sanger sequencing subsequently verified that these aberrant transcripts were attributable to exon 2 skipping, and retention of an 8-bp intronic segment immediately upstream of the native S3 acceptor site, respectively (**Figure 5C**). Mechanistically, both splicing events disrupt the open reading frame (ORF) and introduce premature termination codons (PTCs), with exon 2 skipping expected to yield p.Gly10ValfsTer5 and the 8-bp insertion predicted to generate p.Gly10ValfsTer14. However, based on established PTC-NMD recognition principles, transcripts harboring such PTCs are predicted to be degraded by nonsense-mediated mRNA decay (NMD) *in vivo (*41). Given that our minigene assay only measures steady-state mRNA levels, it does not provide direct experimental evidence for NMD engagement. Nevertheless, even if these aberrant transcripts escape NMD surveillance *in vivo*, the resulting truncated glycosyltransferase polypeptides would lack the catalytic domain and thus be catalytically inactive.

Notably, despite the complete absence of normally spliced transcripts in the minigene system, the three variants nevertheless yield positive absorption-elution results, indicating residual antigen expression *in vivo*, consistent with recent findings by Zhang et al. on splice-site variants of the ABO*B.01 allele (30). This discrepancy implies that the minigene assay may not faithfully mirror the actual splicing events occurring *in vivo*. From the perspective of molecular regulatory architecture, the minigene construct retains only limited intronic and exonic sequences and thus lacks multiple distal regulatory elements of the native ABO locus, including splicing enhancers/silencers, Alu repeats, and the +5.8-kb erythroid-specific transcriptional enhancer (42–44). Some of these elements orchestrate pre-mRNA splice-site recognition (45), whereas others mediate tissue-specific ABO transcription (46). Loss of these elements in the minigene construct may exacerbate the splicing disruption conferred by the variants and compromise functional transcript production. *In vitro*, minigene-transfected cell lines lack the splicing factor repertoire, including serine/arginine-rich proteins (SR proteins) and heterogeneous nuclear ribonucleoproteins (hnRNPs), as well as the transcription factor landscape, including GATA-binding factor 1 (GATA-1) and runt-related transcription factor 1 (RUNX1), that are characteristic of erythroid progenitors at specific differentiation stages. These two factor classes operate at distinct levels, namely splicing and transcriptional control, and erythroid differentiation entails a developmental stage-specific splicing regulatory network (47, 48), wherein compositional and abundance variations of cell-type-specific splicing factors profoundly affect splice-site usage. The absence of such factor combinations in non-erythroid cells may partially account for the discordance between *in vitro* splicing products and *in vivo* phenotypes. Lin et al. reported that splice-site variants yield discrepant splicing product spectra between minigene and full-length gene constructs, especially in detecting low-abundance correctly spliced transcripts (49). Thus, the intact regulatory network and cell-type-specific splicing factor milieu *in vivo* may partially offset splice-site variant-induced functional impairment, permitting low-frequency correct splicing. Synthesizing these lines of evidence, we postulate that the native 3’SS is not completely disrupted *in vivo*, permitting the production of extremely low-abundance correctly spliced transcripts that translate into minute amounts of glycosyltransferase and give rise to extremely weak blood group antigen expression. Another possible mechanism involves translation initiation from non-AUG start codons, which may generate N-terminally truncated GTA proteins that retain part of the C-terminal catalytic domain, thereby preserving residual enzymatic activity (50–52). However, we detected no evidence of normal transcripts or non-AUG translation initiation products in our experimental system. Furthermore, fresh blood samples from the proband were unavailable for RT-PCR analysis of endogenous ABO transcripts, which would have provided direct evidence of *in vivo* splicing products and helped clarify the discrepancy. This limitation precludes a definitive resolution of whether the residual antigen expression originates from extremely low-abundance correctly spliced transcripts or from translation initiation at non-AUG codons. Thus, the origin of functional glycosyltransferases in these variants remains undetermined and merits further experimental validation.

The three variants converged on a common aberrant splicing pattern and absorption-elution phenotype, suggesting that they may operate through shared regulatory mechanisms. Mutational tolerance near splice sites is determined by both distance and nucleotide identity, and is further modulated by cryptic splice-site activation and dynamic splicing factor competition (53, 54). To dissect this, we performed saturation mutagenesis coupled with in silico prediction across positions 1–20 upstream of the *ABO* intron 1 3’SS, and delineated four principles: (i) exon 2 skipping was the sole common aberrant event shared by all splicing-disruptive variants, identifying it as the predominant molecular mechanism underlying ABO subtype(**Table 3**); (ii) the AG dinucleotide at positions 1 and 2 was functionally irreplaceable, as any substitution nearly abolished 3’SS recognition, reinforcing AG as an absolutely conserved core element (55); position 3 was relatively conserved, with substitutions to A or G markedly reducing stability, whereas positions 4 and 15 exhibited high mutational tolerance, with all substitutions preserving normal splicing (**Figure 2**). (iii) within the 5 to 20 region, the impact of base substitutions on splice-site stability followed a strict chemical class-dependent hierarchy, wherein pyrimidines (T/C) conferred markedly higher stability than purines (A/G), with C>T being the least disruptive and T>G posing the highest risk (**Figure 2**); and (iv) at positions with a wild-type T, a distinct position-dependent distance effect was observed within the 5 to 20 region (**Figure 6**). Both T>G and T>A scores were positively correlated with distance, indicating that increased distance correlates with reduced splicing disruption.

Synthesizing these findings, we propose an initial position- and base-dependent risk stratification model. Spatially, positions 1 to 3 confer the highest disruptive potential, while positions 4 and 15 are relatively stable; within the 5 to 20 region, proximity to the 3’SS correlates positively with risk. With respect to base identity, substitutions to G or A confer higher risk, followed by C, with T being least deleterious; among all substitutions, T>G poses the highest risk, whereas C>T exerts the minimal impact. This model translates the relationship between position and base type into simple rules that can guide clinical decisions, allowing initial risk assessment using only the variation’s location and the specific base change.

This study has two main limitations. First, fresh blood samples were unavailable, precluding RT-PCR analysis of endogenous ABO transcripts and flow cytometric quantification of An antigen density. Second, the base- and distance-dependent patterns at the 3’SS were derived primarily from computational predictions; although three variants were experimentally validated, the dataset is too small to draw definitive conclusions. Therefore, the generalizability of the model is limited, and further validation using additional variants and functional assays is required.

## 5 Conclusion

Here, we identified a novel intronic 3’SS variant, c.29-2A>G, in a Chinese A_el_ individual carrying an ABO*A1.02 allele. The nucleotide sequence of this allele has been deposited in GenBank under accession number PZ263082. Functional analysis of this variant together with two previously reported variations, c.29-3C>G and c.29-10T>A, showed that all three disrupt 3’ splice site function, leading to aberrant splicing predominantly via exon 2 skipping, with c.29-10T>A additionally activating a cryptic acceptor site to generate a secondary transcript with an 8-bp intronic insertion. Through computational saturation mutagenesis, we further demonstrate that exon 2 skipping is the predominant splicing defect conferred by these 3’SS mutations, and propose an initial position- and base-dependent risk stratification model that converts base identity and acceptor-site proximity to a quantitative splicing risk score for clinical interpretation. Collectively, these findings provide the first *in vitro* evidence linking 3’SS variants to ABO subtype pathogenesis, point to exon 2 skipping as a core shared molecular pathway, and offer a preliminary computational derived framework for interpreting 3’SS variants in blood grouping and transfusion compatibility assessment, with implications that may extend to splice-site variant interpretation in other genetic systems.

## Data Availability

The datasets presented in this study can be found in online repositories. The names of the repository/repositories and accession number(s) can be found below: https://www.ncbi.nlm.nih.gov/genbank/, PZ263082.

## Supplementary material

Supplementary Figure 1 Splicing patterns of WT and three variants. WT exhibited normal splicing, while all three variants promoted exon 2 skipping, with the c.29-10T>A variant additionally activating a cryptic acceptor site.
